# Dichlorido[(*R*,*R*)-*N* ^1^,*N* ^1^,*N* ^2^-tribenzyl­cyclo­hexane-1,2-diamine-κ^2^ *N* ^1^,*N* ^2^]copper(II)

**DOI:** 10.1107/S1600536810014054

**Published:** 2010-04-24

**Authors:** Quang Trung Nguyen, Jong Hwa Jeong

**Affiliations:** a Department of Chemistry, Kyungpook National University, Taegu 702-701, Republic of Korea

## Abstract

In the title compound, [CuCl_2_(C_27_H_32_N_2_)], which bears a chiral diamine ligand, *viz* (*R*,*R*)-*N*,*N*,*N*′′- tribenzyl­cyclo­hexane-1,2-diamine, the Cu^II^ ion is ligated by two N and two Cl atoms in a distorted square-planar geometry. The coordination of the ligands to the Cu^II^ ion results in the formation of a five-membered heterocyclic ring and a chiral center at the monosubstituted nitro­gen in an (*S*)-configuration. The catalytic capacity of the complex for the asymmetric nitro­aldol reaction is promising (49% ee).

## Related literature

For the synthesis of *N*,*N*,*N*′′-tribenzyl-(*R*,*R*)-1,2-diamino­cyclo­hexane, see: Tye *et al.* (2002 ▶); Boyd *et al.* (2005 ▶). For related structures, see: Alexakis *et al.* (2001 ▶); Tye *et al.* (2002 ▶); Boyd *et al.* (2005 ▶, 2006 ▶); Arjan *et al.* (2005 ▶); Brethon *et al.* (2004 ▶); Jones & Mahon (2008 ▶); Evans & Seidel (2005 ▶); Evans *et al.* (2007 ▶); Roh *et al.* (2004 ▶); Nguyen & Jeong (2008*a* ▶,*b* ▶).
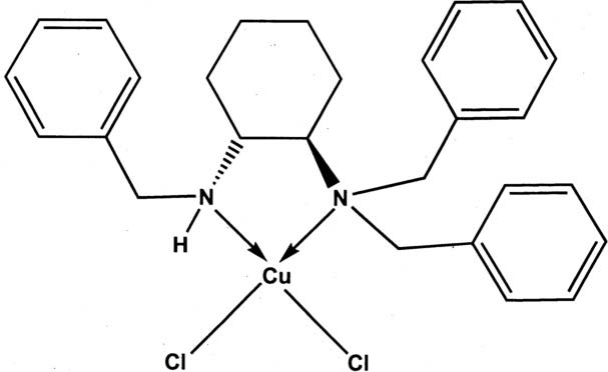


## Experimental

### 

#### Crystal data

[CuCl_2_(C_27_H_32_N_2_)]
*M* *_r_* = 519.00
Orthorhombic, 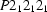

*a* = 10.5806 (7) Å
*b* = 15.4409 (8) Å
*c* = 16.2579 (12) Å
*V* = 2656.1 (3) Å^3^
*Z* = 4
Mo *K*α radiation
μ = 1.04 mm^−1^
*T* = 295 K
0.40 × 0.40 × 0.40 mm

#### Data collection

Enraf–Nonius CAD-4 diffractometer
Absorption correction: analytical (*ABSCALC*; McArdle & Daly, 1999 ▶) *T*_min_ = 0.660, *T*_max_ = 0.666
5793 measured reflections
4931 independent reflections
3885 reflections with *I* > 2σ(*I*)
*R*_int_ = 0.019
3 standard reflections every 60 min  intensity decay: none

#### Refinement

*R*[*F* ^2^ > 2σ(*F* ^2^)] = 0.031
*wR*(*F* ^2^) = 0.083
*S* = 1.06
4931 reflections
292 parameters
H atoms treated by a mixture of independent and constrained refinement
Δρ_max_ = 0.33 e Å^−3^
Δρ_min_ = −0.24 e Å^−3^
Absolute structure: Flack (1983 ▶)
Flack parameter: −0.017 (13)

### 

Data collection: *CAD4* (Enraf–Nonius, 1989 ▶); cell refinement: *CAD4*; data reduction: *XCAD* (McArdle, 1999 ▶); program(s) used to solve structure: *SHELXS97* (Sheldrick, 2008 ▶); program(s) used to refine structure: *SHELXL97* (Sheldrick, 2008 ▶); molecular graphics: *ORTEPIII* (Burnett & Johnson, 1996 ▶); software used to prepare material for publication: *WinGX* (Farrugia, 1999 ▶).

## Supplementary Material

Crystal structure: contains datablocks global, I. DOI: 10.1107/S1600536810014054/rk2198sup1.cif

Structure factors: contains datablocks I. DOI: 10.1107/S1600536810014054/rk2198Isup2.hkl

Additional supplementary materials:  crystallographic information; 3D view; checkCIF report

## Figures and Tables

**Fig. 1.**
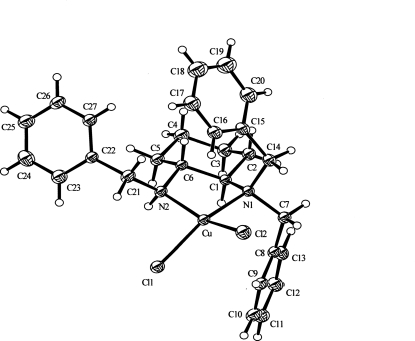
A view of title compound molecule with the atom numbering scheme. Displacement ellipsoids are drawn at the 30% probability level. Hydrogen atoms are presented as a small spheres of arbitrary radius.

## supplementary crystallographic information

### Comment

Disubstituted, trisubstituted and tetrasubstituted (*R*,*R*)-1,2- diaminocyclohexane were synthesized (Alexakis *et al.*, 2001; Tye *et al.*, 2002; Boyd *et al.*, 2005, 2006; Arjan *et al.*, 2005). Especially disubstituted chiral diamine ligands with Rh (Brethon *et al.*, 2004; Jones & Mahon, 2008), Ni (Evans & Seidel, 2005; Evans *et al.*, 2007), Zn (Roh *et al.*, 2004; Nguyen & Jeong, 2008a), Cu (Nguyen & Jeong, 2008b) were extensively applied in asymmetric synthesis. However, the coordination chemistry and application of asymmetric trisubstituted chiral 1,2-diaminocyclohexanes containing a secondary and a tertiary amines had not attended much. In this study, a new complex of Cu(II) containing *N*,*N*,*N'*-tribenzyl-(*R*,*R*)-1,2- diaminocyclohexane (Tye *et al.*, 2002; Boyd *et al.*, 2005) was synthesized and its molecular and crystal structures were determined.

Also, capability of the complex as an enantioselective catalyst for asymmetric nitroaldol reaction was examined. The copper ion was ligated by two nitrogen and two chloride atoms in distorted square-planar geometry. The coordination of the ligands to the Cu ion induced a 5–membered heterocyclic ring and a chiral center at monosubstituted nitrogen in (*S*)-configuration. Catalytic capacity of the complex for asymmetric nitroaldol reaction was promising (49% ee  {ee = [R - S/ R+S] x 100  or [S - R/ R+S] x 100}).

### Experimental

A solution of *N*,*N*,*N'*-tribenzyl-(*R*,*R*)- 1,2-diaminocyclohexane (1.57 g, 4.08 mmol) in ethanol (5 ml) was added slowly to a solution of CuCl_2_^.^2H_2_O (0.69 g, 4.01 mmol) in ethanol (10 ml) Tye *et al.*, (2002); Boyd *et al.*, (2005). The mixture was stirred overnight at ambient temperature. The solvent was removed to yield blue solids. The product was re–crystallized from anhydrous ethanol to afford blue crystals (1.64 g, yield 79%). Anal. Calc. for C_27_H_32_Cl_2_CuN_2_: C 62.48, H 6.21, N 5.40 and found: C 62.20, H 6.30, N 5.46%.

### Refinement

H–atom of N—H was refined with U_iso_(H) = 1.2U_eq_(N). All H–atoms placed on C atoms were positioned geometrically and refined using a riding model with C—H = 0.97Å for methylene, C—H = 0.98Å for methine, C—H = 0.93Å for aromatic H atoms. For all H atoms *U*_iso_(H) = 1.2*U*_eq_(C).

In the crystal structure was found 'accessible void' with volume 54.00Å^3^.

### Crystal data

**Table:**

[CuCl_2_(C_27_H_32_N_2_)]	*F*(000) = 1084
*M**_r_* = 519.00	*D*_x_ = 1.298 Mg m^−^^3^
Orthorhombic, *P*2_1_2_1_2_1_	Mo *K*α radiation, λ = 0.71073 Å
Hall symbol: P 2ac 2ab	Cell parameters from 25 reflections
*a* = 10.5806 (7) Å	θ = 10–13°
*b* = 15.4409 (8) Å	µ = 1.04 mm^−^^1^
*c* = 16.2579 (12) Å	*T* = 295 K
*V* = 2656.1 (3) Å^3^	Block, blue
*Z* = 4	0.40 × 0.40 × 0.40 mm

### Data collection

**Table:**

Enraf–Nonius CAD-4 diffractometer	3885 reflections with *I* > 2σ(*I*)
Radiation source: fine-focus sealed tube	*R*_int_ = 0.019
graphite	θ_max_ = 25.5°, θ_min_ = 1.8°
ω/2θ scans	*h* = −12→12
Absorption correction: analytical (*ABSCALC*; McArdle & Daly, 1999)	*k* = −18→18
*T*_min_ = 0.660, *T*_max_ = 0.666	*l* = −19→19
5793 measured reflections	3 standard reflections every 60 min
4931 independent reflections	intensity decay: none

### Refinement

**Table:**

Refinement on *F*^2^	Secondary atom site location: difference Fourier map
Least-squares matrix: full	Hydrogen site location: inferred from neighbouring sites
*R*[*F*^2^ > 2σ(*F*^2^)] = 0.031	H atoms treated by a mixture of independent and constrained refinement
*wR*(*F*^2^) = 0.083	*w* = 1/[σ^2^(*F*_o_^2^) + (0.0488*P*)^2^] where *P* = (*F*_o_^2^ + 2*F*_c_^2^)/3
*S* = 1.06	(Δ/σ)_max_ = 0.001
4931 reflections	Δρ_max_ = 0.33 e Å^−^^3^
292 parameters	Δρ_min_ = −0.24 e Å^−^^3^
0 restraints	Absolute structure: Flack (1983)
Primary atom site location: structure-invariant direct methods	Flack parameter: −0.017 (13)

### Special details

**Table:**

Geometry. All s.u.'s (except the s.u. in the dihedral angle between two l.s. planes) are estimated using the full covariance matrix. The cell s.u.'s are taken into account individually in the estimation of s.u.'s in distances, angles and torsion angles; correlations between s.u.'s in cell parameters are only used when they are defined by crystal symmetry. An approximate (isotropic) treatment of cell s.u.'s is used for estimating s.u.'s involving l.s. planes.

### Fractional atomic coordinates and isotropic or equivalent isotropic displacement parameters (Å^2^)

**Table:**

	*x*	*y*	*z*	*U*_iso_*/*U*_eq_	
Cu	0.03338 (3)	0.43742 (2)	0.76295 (2)	0.03853 (10)	
Cl1	−0.03484 (8)	0.33981 (5)	0.85529 (5)	0.05231 (19)	
Cl2	−0.10262 (9)	0.40812 (6)	0.66326 (6)	0.0609 (2)	
N1	0.0744 (2)	0.56146 (15)	0.72591 (14)	0.0361 (5)	
N2	0.2032 (2)	0.43863 (17)	0.81961 (17)	0.0403 (5)	
H2	0.193 (3)	0.438 (2)	0.871 (2)	0.048*	
C1	0.1701 (3)	0.59164 (18)	0.78886 (17)	0.0352 (6)	
H1	0.1234	0.5982	0.8406	0.042*	
C2	0.2323 (3)	0.67928 (19)	0.7726 (2)	0.0476 (7)	
H2A	0.2862	0.6755	0.7245	0.057*	
H2B	0.1679	0.7226	0.7621	0.057*	
C3	0.3099 (4)	0.7054 (2)	0.8468 (2)	0.0561 (9)	
H3A	0.3490	0.7612	0.8368	0.067*	
H3B	0.2552	0.7108	0.8944	0.067*	
C4	0.4109 (3)	0.6388 (2)	0.8639 (2)	0.0602 (9)	
H4A	0.4581	0.6556	0.9125	0.072*	
H4B	0.4692	0.6366	0.8179	0.072*	
C5	0.3532 (3)	0.5492 (2)	0.8772 (2)	0.0489 (8)	
H5A	0.3028	0.5498	0.9271	0.059*	
H5B	0.4204	0.5072	0.8845	0.059*	
C6	0.2699 (3)	0.52161 (18)	0.8048 (2)	0.0373 (7)	
H6	0.3229	0.5156	0.7557	0.045*	
C7	−0.0463 (3)	0.61569 (18)	0.73180 (19)	0.0433 (7)	
H7A	−0.0267	0.6739	0.7135	0.052*	
H7B	−0.1075	0.5919	0.6936	0.052*	
C8	−0.1073 (3)	0.62181 (19)	0.8143 (2)	0.0428 (7)	
C9	−0.1934 (3)	0.5605 (2)	0.8410 (2)	0.0544 (8)	
H9	−0.2095	0.5122	0.8085	0.065*	
C10	−0.2553 (3)	0.5697 (3)	0.9143 (3)	0.0645 (10)	
H10	−0.3108	0.5267	0.9317	0.077*	
C11	−0.2371 (4)	0.6404 (3)	0.9621 (2)	0.0628 (10)	
H11	−0.2812	0.6469	1.0112	0.075*	
C12	−0.1515 (4)	0.7031 (3)	0.9366 (2)	0.0609 (10)	
H12	−0.1373	0.7517	0.9692	0.073*	
C13	−0.0880 (4)	0.6938 (2)	0.8638 (2)	0.0530 (9)	
H13	−0.0312	0.7364	0.8473	0.064*	
C14	0.1132 (3)	0.5719 (2)	0.63786 (17)	0.0465 (7)	
H14A	0.1232	0.6333	0.6273	0.056*	
H14B	0.0438	0.5518	0.6038	0.056*	
C15	0.2318 (3)	0.5269 (2)	0.60886 (19)	0.0502 (8)	
C16	0.2368 (4)	0.4375 (3)	0.59948 (19)	0.0564 (8)	
H16	0.1669	0.4039	0.6127	0.068*	
C17	0.3453 (4)	0.3988 (3)	0.5705 (3)	0.0759 (12)	
H17	0.3495	0.3388	0.5664	0.091*	
C18	0.4472 (5)	0.4482 (4)	0.5478 (3)	0.1003 (17)	
H18	0.5202	0.4216	0.5284	0.120*	
C19	0.4416 (5)	0.5367 (4)	0.5536 (3)	0.0976 (17)	
H19	0.5098	0.5703	0.5368	0.117*	
C20	0.3350 (4)	0.5754 (3)	0.5844 (2)	0.0744 (12)	
H20	0.3320	0.6354	0.5888	0.089*	
C21	0.2727 (3)	0.3570 (2)	0.7986 (2)	0.0551 (9)	
H21A	0.2184	0.3082	0.8114	0.066*	
H21B	0.2874	0.3562	0.7397	0.066*	
C22	0.3962 (3)	0.34379 (19)	0.8410 (2)	0.0432 (7)	
C23	0.4030 (4)	0.3147 (3)	0.9210 (2)	0.0631 (10)	
H23	0.3292	0.3016	0.9495	0.076*	
C24	0.5196 (5)	0.3048 (2)	0.9596 (2)	0.0737 (12)	
H24	0.5238	0.2849	1.0135	0.088*	
C25	0.6279 (4)	0.3247 (3)	0.9178 (3)	0.0705 (12)	
H25	0.7056	0.3197	0.9440	0.085*	
C26	0.6229 (3)	0.3515 (2)	0.8387 (3)	0.0621 (10)	
H26	0.6970	0.3628	0.8099	0.075*	
C27	0.5094 (3)	0.3617 (2)	0.8020 (2)	0.0517 (8)	
H27	0.5073	0.3816	0.7480	0.062*	

### Atomic displacement parameters (Å^2^)

**Table:**

	*U*^11^	*U*^22^	*U*^33^	*U*^12^	*U*^13^	*U*^23^
Cu	0.03398 (17)	0.03603 (16)	0.04556 (19)	0.00039 (16)	−0.00646 (17)	−0.00163 (15)
Cl1	0.0463 (4)	0.0478 (4)	0.0629 (5)	−0.0078 (4)	−0.0052 (4)	0.0079 (3)
Cl2	0.0622 (5)	0.0594 (5)	0.0612 (5)	−0.0084 (4)	−0.0245 (4)	−0.0054 (4)
N1	0.0349 (11)	0.0387 (11)	0.0348 (12)	0.0045 (10)	−0.0041 (9)	−0.0005 (11)
N2	0.0347 (12)	0.0368 (12)	0.0494 (14)	0.0047 (12)	−0.0050 (11)	−0.0005 (13)
C1	0.0369 (15)	0.0370 (15)	0.0317 (15)	0.0021 (12)	−0.0037 (12)	−0.0027 (11)
C2	0.0491 (17)	0.0375 (15)	0.0563 (19)	−0.0031 (13)	−0.0058 (16)	−0.0004 (15)
C3	0.060 (2)	0.0404 (18)	0.068 (2)	−0.0031 (16)	−0.0085 (19)	−0.0075 (16)
C4	0.0491 (19)	0.056 (2)	0.075 (2)	−0.0027 (17)	−0.0152 (18)	−0.0130 (18)
C5	0.0405 (17)	0.046 (2)	0.060 (2)	0.0053 (14)	−0.0171 (15)	−0.0075 (15)
C6	0.0308 (15)	0.0376 (15)	0.0435 (17)	0.0020 (13)	−0.0003 (13)	−0.0048 (13)
C7	0.0416 (16)	0.0432 (15)	0.0452 (15)	0.0113 (13)	−0.0069 (16)	0.0001 (13)
C8	0.0360 (16)	0.0383 (16)	0.0540 (19)	0.0081 (14)	−0.0024 (15)	0.0009 (14)
C9	0.0398 (17)	0.0482 (18)	0.075 (2)	−0.0005 (17)	−0.0008 (17)	−0.0117 (19)
C10	0.0439 (19)	0.062 (2)	0.087 (3)	−0.0029 (19)	0.0155 (18)	0.007 (2)
C11	0.058 (2)	0.071 (3)	0.060 (2)	0.016 (2)	0.0131 (18)	0.004 (2)
C12	0.070 (2)	0.055 (2)	0.057 (2)	0.0071 (19)	0.0078 (19)	−0.0099 (17)
C13	0.061 (2)	0.0385 (17)	0.059 (2)	0.0054 (15)	0.0057 (17)	0.0001 (15)
C14	0.0547 (18)	0.0492 (18)	0.0357 (15)	0.0029 (17)	0.0005 (14)	0.0012 (14)
C15	0.061 (2)	0.058 (2)	0.0313 (16)	−0.0050 (17)	0.0085 (15)	−0.0060 (14)
C16	0.065 (2)	0.061 (2)	0.0434 (18)	−0.002 (2)	0.0110 (15)	−0.0125 (18)
C17	0.081 (3)	0.075 (3)	0.072 (3)	0.008 (2)	0.012 (2)	−0.027 (2)
C18	0.075 (3)	0.127 (4)	0.099 (3)	0.005 (3)	0.036 (3)	−0.035 (3)
C19	0.082 (3)	0.115 (4)	0.096 (3)	−0.022 (3)	0.047 (3)	−0.017 (3)
C20	0.085 (3)	0.076 (3)	0.062 (2)	−0.015 (2)	0.029 (2)	−0.006 (2)
C21	0.0438 (19)	0.0413 (18)	0.080 (2)	0.0136 (15)	−0.0093 (18)	−0.0090 (17)
C22	0.0367 (16)	0.0350 (15)	0.058 (2)	0.0057 (13)	0.0038 (15)	0.0010 (14)
C23	0.059 (2)	0.061 (2)	0.070 (3)	0.0144 (19)	0.020 (2)	0.0199 (19)
C24	0.096 (3)	0.071 (3)	0.055 (2)	0.030 (3)	−0.005 (2)	0.0129 (18)
C25	0.054 (2)	0.071 (3)	0.086 (3)	0.019 (2)	−0.017 (2)	−0.008 (2)
C26	0.0404 (19)	0.050 (2)	0.096 (3)	0.0027 (16)	0.008 (2)	−0.001 (2)
C27	0.046 (2)	0.0439 (17)	0.065 (2)	0.0104 (14)	0.0076 (16)	0.0028 (15)

### Geometric parameters (Å, °)

**Table:**

Cu—N2	2.019 (2)	C10—H10	0.9300
Cu—N1	2.054 (2)	C11—C12	1.389 (5)
Cu—Cl2	2.2141 (9)	C11—H11	0.9300
Cu—Cl1	2.2463 (8)	C12—C13	1.369 (5)
N1—C14	1.498 (4)	C12—H12	0.9300
N1—C1	1.513 (3)	C13—H13	0.9300
N1—C7	1.530 (3)	C14—C15	1.511 (5)
N2—C6	1.482 (4)	C14—H14A	0.9700
N2—C21	1.499 (4)	C14—H14B	0.9700
N2—H2	0.85 (3)	C15—C20	1.383 (5)
C1—C2	1.528 (4)	C15—C16	1.389 (5)
C1—C6	1.533 (4)	C16—C17	1.377 (5)
C1—H1	0.9800	C16—H16	0.9300
C2—C3	1.513 (5)	C17—C18	1.371 (6)
C2—H2A	0.9700	C17—H17	0.9300
C2—H2B	0.9700	C18—C19	1.371 (7)
C3—C4	1.509 (5)	C18—H18	0.9300
C3—H3A	0.9700	C19—C20	1.371 (6)
C3—H3B	0.9700	C19—H19	0.9300
C4—C5	1.528 (5)	C20—H20	0.9300
C4—H4A	0.9700	C21—C22	1.492 (5)
C4—H4B	0.9700	C21—H21A	0.9700
C5—C6	1.531 (4)	C21—H21B	0.9700
C5—H5A	0.9700	C22—C23	1.379 (5)
C5—H5B	0.9700	C22—C27	1.383 (4)
C6—H6	0.9800	C23—C24	1.392 (6)
C7—C8	1.492 (4)	C23—H23	0.9300
C7—H7A	0.9700	C24—C25	1.366 (6)
C7—H7B	0.9700	C24—H24	0.9300
C8—C9	1.383 (5)	C25—C26	1.352 (6)
C8—C13	1.388 (5)	C25—H25	0.9300
C9—C10	1.368 (5)	C26—C27	1.350 (5)
C9—H9	0.9300	C26—H26	0.9300
C10—C11	1.354 (6)	C27—H27	0.9300
			
N2—Cu—N1	86.39 (10)	C10—C9—C8	121.2 (4)
N2—Cu—Cl2	156.09 (8)	C10—C9—H9	119.4
N1—Cu—Cl2	96.50 (7)	C8—C9—H9	119.4
N2—Cu—Cl1	89.27 (8)	C11—C10—C9	121.1 (4)
N1—Cu—Cl1	152.80 (7)	C11—C10—H10	119.5
Cl2—Cu—Cl1	98.24 (4)	C9—C10—H10	119.5
C14—N1—C1	115.5 (2)	C10—C11—C12	118.9 (4)
C14—N1—C7	103.3 (2)	C10—C11—H11	120.5
C1—N1—C7	110.3 (2)	C12—C11—H11	120.5
C14—N1—Cu	115.99 (19)	C13—C12—C11	120.4 (4)
C1—N1—Cu	103.32 (16)	C13—C12—H12	119.8
C7—N1—Cu	108.40 (17)	C11—C12—H12	119.8
C6—N2—C21	117.2 (2)	C12—C13—C8	120.9 (3)
C6—N2—Cu	110.95 (18)	C12—C13—H13	119.6
C21—N2—Cu	108.9 (2)	C8—C13—H13	119.6
C6—N2—H2	103 (2)	N1—C14—C15	118.5 (3)
C21—N2—H2	106 (2)	N1—C14—H14A	107.7
Cu—N2—H2	110 (2)	C15—C14—H14A	107.7
N1—C1—C2	116.4 (2)	N1—C14—H14B	107.7
N1—C1—C6	111.0 (2)	C15—C14—H14B	107.7
C2—C1—C6	111.0 (2)	H14A—C14—H14B	107.1
N1—C1—H1	105.9	C20—C15—C16	118.5 (3)
C2—C1—H1	105.9	C20—C15—C14	119.8 (3)
C6—C1—H1	105.9	C16—C15—C14	121.5 (3)
C3—C2—C1	109.4 (3)	C17—C16—C15	120.0 (4)
C3—C2—H2A	109.8	C17—C16—H16	120.0
C1—C2—H2A	109.8	C15—C16—H16	120.0
C3—C2—H2B	109.8	C18—C17—C16	120.4 (4)
C1—C2—H2B	109.8	C18—C17—H17	119.8
H2A—C2—H2B	108.2	C16—C17—H17	119.8
C4—C3—C2	110.5 (3)	C19—C18—C17	120.1 (5)
C4—C3—H3A	109.6	C19—C18—H18	119.9
C2—C3—H3A	109.6	C17—C18—H18	119.9
C4—C3—H3B	109.6	C18—C19—C20	119.7 (5)
C2—C3—H3B	109.6	C18—C19—H19	120.2
H3A—C3—H3B	108.1	C20—C19—H19	120.2
C3—C4—C5	111.1 (3)	C19—C20—C15	121.2 (4)
C3—C4—H4A	109.4	C19—C20—H20	119.4
C5—C4—H4A	109.4	C15—C20—H20	119.4
C3—C4—H4B	109.4	C22—C21—N2	116.0 (3)
C5—C4—H4B	109.4	C22—C21—H21A	108.3
H4A—C4—H4B	108.0	N2—C21—H21A	108.3
C4—C5—C6	111.9 (3)	C22—C21—H21B	108.3
C4—C5—H5A	109.2	N2—C21—H21B	108.3
C6—C5—H5A	109.2	H21A—C21—H21B	107.4
C4—C5—H5B	109.2	C23—C22—C27	116.9 (3)
C6—C5—H5B	109.2	C23—C22—C21	121.8 (3)
H5A—C5—H5B	107.9	C27—C22—C21	121.3 (3)
N2—C6—C5	112.9 (3)	C22—C23—C24	120.5 (4)
N2—C6—C1	108.0 (2)	C22—C23—H23	119.8
C5—C6—C1	109.3 (2)	C24—C23—H23	119.8
N2—C6—H6	108.8	C25—C24—C23	119.6 (3)
C5—C6—H6	108.8	C25—C24—H24	120.2
C1—C6—H6	108.8	C23—C24—H24	120.2
C8—C7—N1	116.9 (2)	C26—C25—C24	120.6 (4)
C8—C7—H7A	108.1	C26—C25—H25	119.7
N1—C7—H7A	108.1	C24—C25—H25	119.7
C8—C7—H7B	108.1	C27—C26—C25	119.4 (4)
N1—C7—H7B	108.1	C27—C26—H26	120.3
H7A—C7—H7B	107.3	C25—C26—H26	120.3
C9—C8—C13	117.6 (3)	C26—C27—C22	123.0 (3)
C9—C8—C7	121.6 (3)	C26—C27—H27	118.5
C13—C8—C7	120.6 (3)	C22—C27—H27	118.5
			
N2—Cu—N1—C14	104.1 (2)	C1—N1—C7—C8	−53.7 (3)
Cl2—Cu—N1—C14	−52.04 (19)	Cu—N1—C7—C8	58.8 (3)
Cl1—Cu—N1—C14	−174.54 (15)	N1—C7—C8—C9	−86.8 (3)
N2—Cu—N1—C1	−23.25 (17)	N1—C7—C8—C13	99.2 (3)
Cl2—Cu—N1—C1	−179.41 (15)	C13—C8—C9—C10	−1.4 (5)
Cl1—Cu—N1—C1	58.1 (2)	C7—C8—C9—C10	−175.5 (3)
N2—Cu—N1—C7	−140.32 (18)	C8—C9—C10—C11	2.0 (6)
Cl2—Cu—N1—C7	63.51 (17)	C9—C10—C11—C12	−1.7 (6)
Cl1—Cu—N1—C7	−59.0 (2)	C10—C11—C12—C13	0.8 (6)
N1—Cu—N2—C6	−1.8 (2)	C11—C12—C13—C8	−0.3 (6)
Cl2—Cu—N2—C6	96.1 (3)	C9—C8—C13—C12	0.5 (5)
Cl1—Cu—N2—C6	−154.94 (19)	C7—C8—C13—C12	174.8 (3)
N1—Cu—N2—C21	−132.2 (2)	C1—N1—C14—C15	59.0 (4)
Cl2—Cu—N2—C21	−34.3 (3)	C7—N1—C14—C15	179.6 (3)
Cl1—Cu—N2—C21	74.7 (2)	Cu—N1—C14—C15	−62.0 (3)
C14—N1—C1—C2	45.3 (3)	N1—C14—C15—C20	−114.2 (4)
C7—N1—C1—C2	−71.3 (3)	N1—C14—C15—C16	71.3 (4)
Cu—N1—C1—C2	173.0 (2)	C20—C15—C16—C17	3.5 (5)
C14—N1—C1—C6	−82.8 (3)	C14—C15—C16—C17	178.0 (3)
C7—N1—C1—C6	160.6 (2)	C15—C16—C17—C18	−2.5 (6)
Cu—N1—C1—C6	44.9 (2)	C16—C17—C18—C19	−0.1 (8)
N1—C1—C2—C3	171.8 (3)	C17—C18—C19—C20	1.7 (8)
C6—C1—C2—C3	−60.1 (3)	C18—C19—C20—C15	−0.7 (8)
C1—C2—C3—C4	59.6 (4)	C16—C15—C20—C19	−1.9 (6)
C2—C3—C4—C5	−57.4 (4)	C14—C15—C20—C19	−176.5 (4)
C3—C4—C5—C6	55.2 (4)	C6—N2—C21—C22	57.9 (4)
C21—N2—C6—C5	−86.4 (3)	Cu—N2—C21—C22	−175.2 (3)
Cu—N2—C6—C5	147.6 (2)	N2—C21—C22—C23	78.9 (4)
C21—N2—C6—C1	152.6 (3)	N2—C21—C22—C27	−100.0 (4)
Cu—N2—C6—C1	26.6 (3)	C27—C22—C23—C24	0.3 (5)
C4—C5—C6—N2	−174.6 (3)	C21—C22—C23—C24	−178.6 (3)
C4—C5—C6—C1	−54.3 (4)	C22—C23—C24—C25	0.4 (6)
N1—C1—C6—N2	−48.7 (3)	C23—C24—C25—C26	−1.8 (6)
C2—C1—C6—N2	−179.7 (2)	C24—C25—C26—C27	2.4 (6)
N1—C1—C6—C5	−171.9 (2)	C25—C26—C27—C22	−1.7 (6)
C2—C1—C6—C5	57.1 (3)	C23—C22—C27—C26	0.3 (5)
C14—N1—C7—C8	−177.7 (3)	C21—C22—C27—C26	179.2 (3)
